# Guidance of visual attention by semantic information in real-world scenes

**DOI:** 10.3389/fpsyg.2014.00054

**Published:** 2014-02-06

**Authors:** Chia-Chien Wu, Farahnaz Ahmed Wick, Marc Pomplun

**Affiliations:** Department of Computer Science, University of MassachusettsBoston, MA, USA

**Keywords:** scene understanding, semantics, attention, scene perception, real-world scenes

## Abstract

Recent research on attentional guidance in real-world scenes has focused on object recognition within the context of a scene. This approach has been valuable for determining some factors that drive the allocation of visual attention and determine visual selection. This article provides a review of experimental work on how different components of context, especially semantic information, affect attentional deployment. We review work from the areas of object recognition, scene perception, and visual search, highlighting recent studies examining semantic structure in real-world scenes. A better understanding on how humans parse scene representations will not only improve current models of visual attention but also advance next-generation computer vision systems and human-computer interfaces.

## INTRODUCTION

For the past two decades, research on the deployment of visual attention has shifted its focus from synthetic stimuli to real-world scenes. Unlike simple statistical structures of synthetic stimuli, real-world environments provide complex layers of information that cannot be processed all at once by our visual system. Despite this overwhelming amount of information, people perform daily visual tasks such as visual search or inspection with only a few glances. Therefore, effective vision greatly depends on the information observers acquire to help them decide where to look next. This has drawn a vast amount of research interest in recent years.

Current models of attentional deployment are divided into two camps – focusing on either top-down (or “endogenous”) mechanisms or bottom-up (or “exogenous”) mechanisms. One of the most influential studies on attentional guidance by bottom-up mechanisms was conducted by Koch and Ullman (1985), proposing the idea of a saliency map. In their model, features of entities in the scene such as edge density, color, intensity, and motion are computed in parallel as by different retinotopic maps in early visual areas. These maps are then combined into a single scalar saliency map representing relative conspicuities across the visual scene. The regions with “high salience” can be used to predict gaze fixation distribution in the scene, which indicates how attention is allocated in a visual scene (Kowler et al., 1995; Findlay, 1997). Therefore, the modeling of bottom-up mechanisms driven by saliency maps has been used extensively to predict the regions where attention is likely to be deployed during natural viewing (e.g., Itti and Koch, 2001; Bruce and Tsotsos, 2009).

**FIGURE 1:**
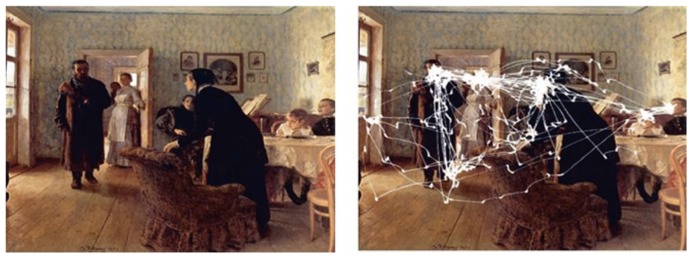
**Left: The oil painting “An Unexpected Visitor” painted by Ilya Repin.** Right: an example of an eye trace measured by Yarbus during the free viewing condition (1967). This figure was originally referred from http://www.cabinetmagazine.org/issues/30/archibald.php.

Though the saliency map serves an important heuristic function in the study of eye movements, predictions from it begin to falter in real-world scenes and often fail to explain how gaze is directed. For example, Yarbus (1967) showed that the effect of context can direct eye movements to important locations in the scene, such as human faces (**Figure 1**). In addition to scene context, the observer’s current task strongly influences allocation of attention. For instance, Hayhoe et al. (2003) asked observers to make a sandwich while their eye and hand movements were recorded. Their results showed that while the participants were performing these tasks in the real environment, they made clusters of fixations only to task-relevant regions. In contrast, task-irrelevant regions, sometimes having higher saliency, were rarely fixated. In order to account for the dominant control of visual attention by top-down mechanisms, many studies have incorporated top-down components into the saliency map to improve the prediction of gaze distribution (see Borji and Itti, 2013, for a review).

**FIGURE 2:**
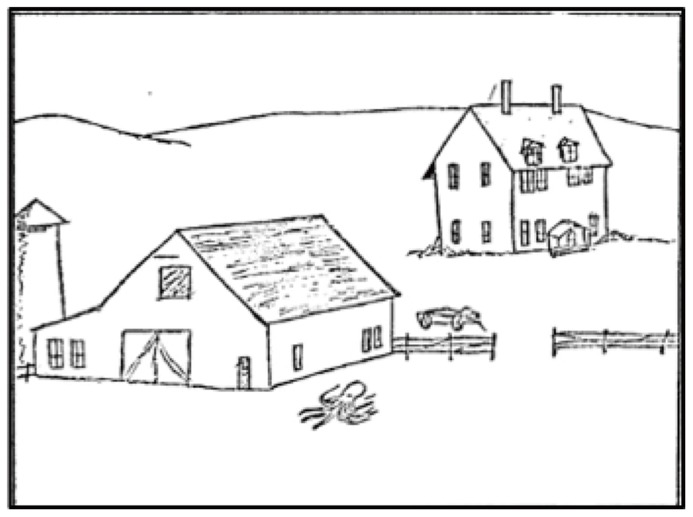
**A stimulus used in Loftus and MacKworth (1978).** A line drawing of an octopus was placed on a line-drawn farmyard. Figure reproduced from Loftus and MacKworth (1978).

Unlike bottom-up mechanisms, which are mainly driven by the physical properties of the scene, top-down mechanisms process visual input in a way that is shaped by the observer’s experience. Top-down mechanisms assign meaning to perceived information based on long-term memory content or knowledge that can be generalized from memory (e.g., inferring that the location of an unknown truck is likely near the ground level). For instance, when observers view the picture used in the study by Loftus and MacKworth (1978), they see “an octopus in a farmyard” rather than “an object at the bottom of the picture” (see **Figure 2**). Top-down mechanisms have been extensively investigated, with a focus on two aspects-one is goal directed, task-driven control (Hayhoe et al., 2003; Jovancevic et al., 2006), and the other is understanding of scene content, that is, how the content of a scene is learned and influences visual behavior (Neider and Zelinsky, 2006; Henderson et al., 2009; Tatler et al., 2010).

There has been a growing interest in the role of semantic information in attentional guidance. In order to access semantic information, the visual input has to be processed in a memory-based manner so that it can be assigned an existing meaning or be associated with a known category. This knowledge-based information has been referred to as “semantic” or “contextual” information by many studies, which have examined scene content at various levels. For example, some research focused on the coarse-level category information of scenes, known as “scene gist” (Friedman, 1979; Schyns and Oliva, 1994; Oliva and Torralba, 2001; Torralba et al., 2006). Other work has examined the relations between scene and object or between objects themselves (Hollingworth and Henderson, 1998; Henderson et al., 1999; Joubert et al., 2007; Võ and Henderson, 2009). Therefore, when prior studies investigated whether and how semantic information in the scene can bias attentional deployment, it was sometimes confusing to discern which type of semantic information was actually being referred to. In order to understand how the visual system uses semantic information provided in the scene and incorporate this knowledge into the current state of attentional models, further clarification is needed.

Previous work has also reviewed semantic information in natural scenes and the role it plays in scene perception (Biederman et al., 1982; Rayner and Pollatsek, 1992; Henderson and Hollingworth, 1998, 1999; Henderson, 2003; Oliva, 2005, 2009; Oliva and Torralba, 2007). It did not, however, directly compare all aspects of semantic information. The current review takes a closer look at the different types of semantic information retrieved from natural scenes and how they may associate with each other. Note that the goal of the current review is not to exhaustively discuss all aspects of semantic information but to provide an overview of some important ongoing debates in this field of research.

Our review is structured as follows: in Section “Contextual Information – the Gist of a Scene,” we summarize research on scene gist, one of the most studied types of semantic information, and discuss how this factor might influence attentional guidance even without accessing the meaning of objects. Section “Scene-Object Relations” examines how different aspects of semantic information can be extracted when meanings of objects are recognized and how the relation between objects and scene may guide visual attention. In Section “Object–Object Relations: Co-Occurrence and Spatial Dependency of Objects,” we further discuss the effects of co-occurrence and spatial dependency between different objects in a scene on the allocation of attention. Section “Conceptual Semantic Associations between Objects in the Scene” discusses how conceptual semantic similarity between objects can be used by the visual system to guide visual attention. In the last section, we summarize the current debates on how different pieces of semantic information are perceived and how they are used to direct attention and facilitate scene understanding. By exploring the different aspects of semantic information, we hope to resolve some inconsistencies in the literature and shed light on how these factors influence visual attention during natural viewing.

## CONTEXTUAL INFORMATION – THE GIST OF A SCENE

What kind of information can people perceive in the early stages of visual processing during natural viewing? Imagine that we are watching TV and rapidly flipping channels from one to another. With only a single glance we can identify the channels we want to skip and have no trouble recognizing whether they are showing news, sports, music, or a movie. This phenomenal ability to recognize each picture with a single glimpse has drawn substantial research interest. Potter (1976) found that, during an RSVP task, observers could detect the target picture, which was pre-specified by a picture or descriptive title, within an exposure as short as 113 ms per image. Potter and Levy (1969) also found that observers were able to memorize an image within a presentation duration of only 100 ms when the image was presented by itself. These results suggest that, during the early visual processing stages, observers can extract many types of basic-level information (such as spatial configuration) and use them to identify the basic content of the scene (such as its category). This information is referred as the “gist” of a scene (Friedman, 1979).

**FIGURE 3:**
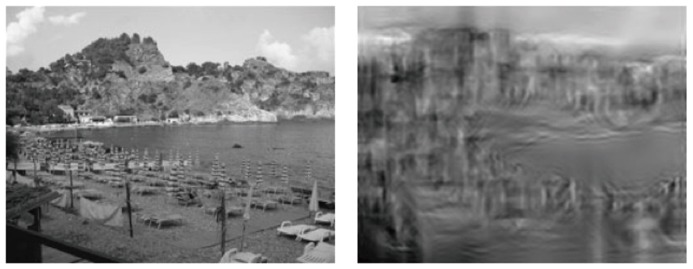
**Illustration of a natural scene (left) and its scene gist (right), generated by an image processing algorithm, that conserves sufficient spatial perceptual dimensions to infer the category of the scene (Figure reproduced from Oliva, 2005, Figure 41.3)**.

Many following studies have investigated how the gist of a scene might affect visual attention and facilitate object recognition. The term “gist” has been widely used to refer to scene content ranging from low-level features (e.g., color or luminance) to high-level information (e.g., events occurring in the scene, see Friedman, 1979). Sometimes it has been termed “scene context” as well, although the context of a scene is often used to refer to co-occurrence relations among objects in the scene (see Section “Object–Object Relations: Co-Occurrence and Spatial Dependency of Objects”). Oliva (2005) provided a brief review about the different levels of scene gist, which includes the gist built during perception (referred to as perceptual gist) and a higher level of gist inferred from more complex semantic information, such as the meaning of an object or the relation between scene and objects (referred to as conceptual gist). According to this distinction, conceptual gist can persist after perceptual information is no longer available. Nevertheless, in the literature, the term “gist of a scene” typically refers to the essential level of information that is able to convey the basic meaning of the scene. For example, Oliva and Torralba (2001) found that humans are sensitive to the spatial structure of natural scenes, which can be used to infer the scene category. **Figure 3** shows an example of how a few global features (contour, density, and color in this case) are sufficient to form the spatial envelope and represent the gist of a scene. Torralba et al. (2006) also found that observers could extract some global scene properties without recognizing individual objects and use this information to guide their attention and eye movements.

Although the studies above have demonstrated that observers are able to use scene gist to facilitate scene understanding, little is known about when and where the gist of a scene is learned. Potter (1976) demonstrated that, within approximately 100 ms, observers were able to not only identify the category of an image, but also recognize some objects and their features. Thorpe et al. (1996) discovered that some meaning of a scene could be understood when it was presented for only 20 ms. These results show that the time course of perceiving the gist is clearly shorter than the time required for object recognition or typical saccadic preparation. This implies a minor role of foveal processing in perceiving a scene gist. A comparable result was also found by Larson and Loschky (2009). They compared observers’ performance in scene recognition between two conditions: a gaze-contingent window condition in which peripheral vision was blocked and only central vision was permitted, and a “scotoma” condition in which central vision was blocked and only peripheral vision was intact. They found that when central vision was blocked, peripheral vision provided sufficient information for recognizing scene gist and performance was unimpaired even when the central “scotoma” was as large as 5°. A similar result was obtained by Boucart et al. (2013). They showed that observers could categorize scenes (highway vs. forest) even at 70° eccentricity. These findings, along with other studies suggesting that scene gist can be extracted from low spatial frequency information alone (Schyns and Oliva, 1994; Oliva and Torralba, 2006), demonstrate that peripheral vision is sufficient for recognizing scene gist.

Since observers can capture the gist of a scene within 100 ms (Potter, 1976) and central vision is not even necessary for gist recognition, this raises the question whether visual attention is required for recognizing scene gist. Li et al. (2002) found that the ability to classify a natural scene presented in peripheral vision is not impaired when the observer is performing a concurrent task presented in the central visual field (known as the dual-task paradigm). This implies that the perception of scene gist may be a “pop-out” preattentive process and does not require focal attention. The ability to detect scene gist instantly even when attention is directed to another task has become a commonly cited evidence for awareness of scene gist without attention (Rensink et al., 1997; Koch and Tsuchiya, 2007; see also Fabre-Thorpe, 2011, for a review). Furthermore, Serre et al. (2007) proposed a hierarchical feed-forward model that made passable predictions of human performance in a categorization task in which each image was only presented for 20 ms. This result shows the neurophysiological plausibility of the rapid categorization task being performed without feedback loops for attentional modulation.

In contrast to these findings, Cohen et al. (2011) argued that the reason prior studies did not find impaired performance for gist detection in the dual-task paradigm was insufficient attentional demand in the central task. They conducted a dual-task experiment with a variety of demanding attention tasks and found that gist detection was impaired when the central task was sufficiently difficult. Their result suggests that awareness of scene gist is not preattentive and attention is essential to the process of perceiving scene gist. In order to reconcile their finding with the demonstrated human ability of extremely fast scene categorization, Cohen et al. (2011) proposed that some components of scene processing may be accomplished preattentively and can bias categorical decisions, whereas actual awareness of scene gist requires at least a small amount of attention. In agreement with this assumption, Kihara and Takeda (2012) found that observers can integrate information from different spatial frequencies without attention.

Regardless of the requirement of attention, the role of object recognition in acquiring the gist is still unclear. As mentioned earlier, some research has claimed that scene gist can be retrieved based on the processing of spatial layout, texture, volume, or other low-level image features and does not depend on recognizing objects (Schyns and Oliva, 1994; Oliva and Torralba, 2001; Torralba et al., 2006). On the other hand, other studies argued that scene gist is not processed independently but can be processed more accurately when the representative or diagnostic object in the scene is recognized. For example, recognizing a reverend in a scene can help in classifying the scene category as a church (Friedman, 1979; Davenport and Potter, 2004; see also Henderson and Hollingworth, 1999, for a detailed review of object recognition and scene context).

To summarize, in the literature on visual attention, scene gist may refer to different types of information provided in the scene. Nevertheless, in most cases the term “gist” indicates the information extracted in early visual processing (20–100 ms) that can convey the meaning of a scene and is sufficient to categorize the scene. Whether object recognition is necessary before perceiving scene gist, or whether visual attention is needed for gist recognition are still open questions.

## SCENE-OBJECT RELATIONS

As discussed above, scene gist may be perceived without recognizing any object in the scene. However, to accomplish the arguably most common visual task – visual search-accessing the meanings of task-related items becomes essential. The gist of a scene not only enables us to recognize the category of environment we are looking at, but also facilitates object recognition, enabling us to locate the most informative region without serially inspecting every single position in a scene (Brockmole et al., 2006; Brockmole and Henderson, 2006a). Visual search in natural scenes is currently a prominent research paradigm because the human visual system still outperforms state-of-the-art computer vision systems. How is this high efficiency achieved? One important factor seems to be that, unlike artificial systems, the human visual system can use the context of a scene to guide attention before most of the scene objects are recognized.

**FIGURE 4:**
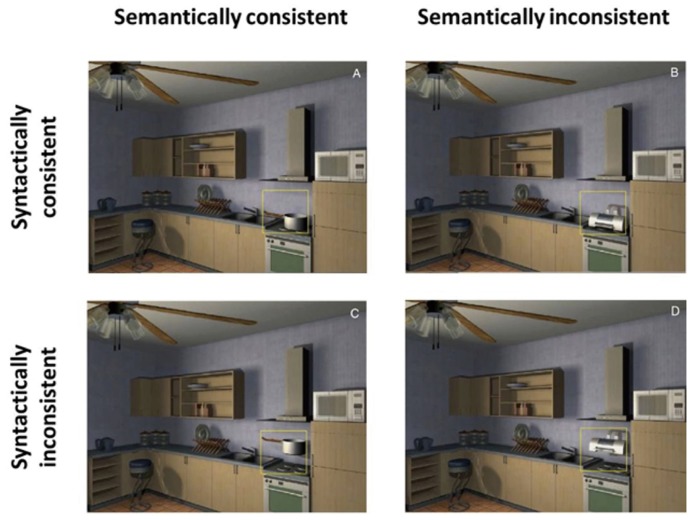
**Examples of semantic and syntactic relations in a kitchen scene.** **(A)** Semantically and syntacticallyconsistent. **(B)** Semantically inconsistent (the printer does not belong in the kitchen scene) but syntactically consistent. **(C)** Semantically consistent but syntactically inconsistent (a floating pot violates gravity). **(D)** Semantically and syntactically inconsistent. Figure reproduced from Võ and Henderson (2009).

When reviewing the literature, we first need to clarify what information is referred to as the “context” of a scene. In addition to scene gist, which may only provide some super ordinate category information about the scene, additional contextual information is conveyed when the meaning of an object is known. Upon recognition of an object, the observer’s visual system considers both its semantic relationship (whether this object’s identity fits in the scene) and spatial relationship within the scene (whether the object’s location is appropriate). Biederman et al. (1982) used the terms “semantic” and “syntactic” to describe the relations between an object and its setting. Semantic relations require access to the object’s meaning and involve probability, position, and expected size of objects in a scene. On the other hand, syntactic relations involve support and interposition, that is, it describes the laws of physics (e.g., whether an object should rest on a surface, or occlude the background). **Figure 4** show examples of semantic and syntactic relations and corresponding violations in a scene.

Scene consistency involving semantic and syntactic relations has been studied in the context of object recognition and visual search. Loftus and MacKworth (1978) investigated how fixations were distributed over consistent and inconsistent objects during picture viewing. In their experiments, participants were asked to memorize line drawings of natural scenes which were composed of “consistent” (e.g., a tractor in a farmyard) versus “inconsistent” (e.g., an octopus in a farmyard, see **Figure 2**) objects. The object congruency violations in their experiments were semantic in nature. Their findings showed that an inconsistent object in the scene was fixated on earlier and for a longer duration during free viewing than a consistent object. They suggested that allocating more attention to inconsistent objects might have been a memorization strategy to distinguish the informative regions in the scene. Some researchers have claimed that these categories (semantic vs. syntactic) are terms of linguistics and do not reflect distinct cognitive signals (see Henderson and Ferreira, 2004). However, a recent EEG study by Võ and Wolfe (2013) found differences in evoked potentials between semantic and syntactic violations during scene perception, indicating that they are being processed in categorically different ways.

A number of studies involving visual search and inspection tasks reported that inconsistent objects in scenes not only drew attention immediately but they also affected early eye movements (Loftus and MacKworth, 1978; Biederman et al., 1982; Hollingworth and Henderson, 1999; Gordon, 2004, 2006; Stirk and Underwood, 2007; Underwood et al., 2007; Bonitz and Gordon, 2008). These findings suggest that object-scene inconsistency attracts attention and gaze without the need for full object identification, also known as the pre-attentive pop-out effect (Johnston et al., 1990; Marks et al., 1992; Brockmole and Henderson, 2008).

Alternatively, many studies have found that consistent objects are easier to detect and identify than inconsistent objects (Boyce and Pollatsek, 1992a,b; De Graef, 1992; Henderson, 1992; Rayner and Pollatsek, 1992; Rensink et al., 1997, 2000; Kelley et al., 2003; Davenport and Potter, 2004; Malcolm and Henderson, 2010). Numerous studies have found that inconsistent object detection was slower and less accurate in scenes containing semantic, syntactic or both violations (Biederman et al., 1982; Henderson et al., 1999). Unlike earlier studies that used line drawings or photographs, Võ and Henderson (2009) used 3D-rendered images of real-world scenes to control for low-level cues. In agreement with the studies mentioned above, they did not find early effects of scene inconsistencies either during scene memorization or visual search. Together these results suggest that the pop-out effects found by previous studies may have been due to inconsistencies in bottom-up saliency such as inconsistent lighting, brightness, shading, or transitions between object and background when violations were created in the stimuli. It is thus possible that attention was drawn to these inconsistencies due to low-level features rather than semantic/syntactic violations in the stimuli.

The main criticisms against pre-attentive pop-out effects are: (1) low-level visual conspicuity of inconsistent objects from the rest of the scene; (2) failure to find fixational precedence for inconsistent over consistent objects regardless of their spatial structure in the scene (Friedman and Liebelt, 1981; Henderson and Hollingworth, 1998); (3) better discrimination performance for consistent extrafoveal objects than for inconsistent ones (De Graef et al., 1990; Hollingworth and Henderson, 1998; Võ and Henderson, 2009). There is more support for the claim that foveal processing is necessary before such inconsistent objects can be detected (Võ and Henderson, 2011) or even affect early eye movements (Henderson et al., 1999; Võ and Henderson, 2009).

How does the background of the scene play a role in deploying attention to objects in that scene? Though Henderson and Hollingworth (1998, 1999) found that objects in scenes were processed independently from their background, Davenport and Potter (2004) proposed an interaction between objects and their background during scene processing. They presented color photographs containing a salient or highly distinctive object in a scene, such as a road with a cyclist. The photographs were presented for 80 ms followed by a mask, and a naming task was used in which subjects were instructed to identify either the object or the background. The results showed that objects were more accurately detected in consistent settings, and backgrounds were perceived more accurately with consistent foreground objects. In another study, Davenport (2007) showed that in addition to the background, objects in scenes exert contextual influences on each other, suggesting that objects and their settings are processed together. Brockmole and Henderson (2006a,b) also found that the association between artificial objects and a natural scene could be learned via repeated exposure and used to facilitate search performance.

A question at this point is how the interplay between visual salience of objects in the scene and their semantic properties affects the allocation of attention during scene perception. The traditional view (Henderson et al., 1999) is that early fixations on a scene are determined by low-level visual features. A number of studies have reported that visual salience plays a dominant role in change detection tasks more than in search tasks (Pringle et al., 2001; Carmi and Itti, 2006; Spotorno and Faure, 2011; Spotorno et al., 2013). When target categories or specific items are altered during change detection tasks, this may impact the visual salience of the objects more than the semantic or syntactic congruency of the stimuli. Kollmorgen et al. (2010) examined the guidance of eye movements during a classification task using three measures: low-level features, high-level task dependent components (e.g., expression or gender of human faces) and spatial bias in stimuli composed of small image patches of either human faces or outdoor scenes. Each of these measures had a significant effect on eye movements. Spatial bias had the strongest effect on the guidance of eye movements. This was closely followed by high-level task-dependent components, and low-level features had less of an impact. The authors also found that task-dependent components had an especially strong effect when categorizing facial expressions. Other findings suggest an interaction between salience and semantic content of the scene (Nyström and Holmqvist, 2008) with semantic content causing more influence over time than visual salience. It is still unclear what proportions of these factors contribute to gaze guidance and how these proportions might vary over viewing time.

Regardless of visual salience or semantic relations between an object and its setting, there are particular classes of objects that can immediately capture our attention in an image. Texts were found to attract more attention than regions with similar size and position in real-world scenes (Cerf et al., 2009; Wang and Pomplun, 2012). Faces also belong to this special category of objects. Yarbus (1967) showed that during free viewing of a painting without any other instruction, fixations of observers were not evenly distributed but clustered around faces of the individuals in the painted scene (see **Figure 1**). In the first study of this kind, Buswell (1935) noted that human figures were disproportionately likely to be fixated on. Recently, Fletcher-Watson et al. (2008) found a similar bias toward human bodies and faces. This attentional preference is not limited to humans but applies to animals as well. In a series of studies, Kirchner and his colleagues (Kirchner et al., 2003; Kirchner and Thorpe, 2006) reported that participants were rapidly, within 120 ms of stimulus onset, able to saccade toward a natural scene with an animal when presented with two such natural images simultaneously.

Computational approaches predicting attentional allocation via fixational distribution performed much better than the traditional saliency map model when detection of special objects such as faces, texts, or both were incorporated. Cerf et al. (2008) integrated the Viola-Jones face detection algorithm (Viola and Jones, 2001) into their saliency model and demonstrated that, with this simple addition, the new model was better at predicting gaze allocation than the original one. Even superior performance was achieved by models using a non-linear combination of several top-down *cognitive* features which affect eye-movements, such as human bodies and interesting objects such as cars, dogs, or computer monitors (see Elazary and Itti, 2008), along with bottom-up features (Borji, 2012; Zhao and Koch, 2012).

To summarize, the majority of findings from the literature suggest that the visual system utilizes knowledge of semantic coherence of a scene during search. This makes detection of inconsistent objects difficult unless these objects violate extreme semantic or syntactic rules. The spatial configuration of objects seems to define the basic structure of a scene and perhaps contributes the most in forming a scene schema.

## OBJECT-OBJECT RELATIONS: CO-OCCURRENCE AND SPATIAL DEPENDENCY OF OBJECTS

The contextual information discussed so far involves either the scene gist or the relation between an object and the background of the scene. However, in the natural environment, objects rarely appear in isolation. The co-occurrence of objects and local spatial layout of the scene can be an alternate conceptualization of scene context (Bar, 2004; Mack and Eckstein, 2011). Object co-occurrence provides information about the likelihood of an object appearing in a scene when a reference object is recognized. For instance, if a scene contains a keyboard, it is likely that there is a mouse next to it. Typically, the concept of co-occurrence is associated with spatial proximity, that is, the tendency of certain objects to be located near each other. 

Only a few studies have investigated the role of object co-occurrence in the deployment of attention in real-world scenes. Mack and Eckstein (2011) investigated the effects of object co-occurrence in natural environments on visual search. They found that viewers searched for targets (e.g., a headphone) at expected locations (e.g., next to an iPod) more efficiently than for targets at unexpected locations (e.g., next to a cup). Furthermore, a disproportionate amount of fixations landed on the relevant cue item (e.g., an iPod when headphones were the search target) as compared to the other scene objects. Similarly, using a flash-preview-moving-window paradigm, Castelhano and Heaven (2011) found that the presence of an object that co-occurs with the target can guide attention and facilitate search even when these pairings are shown on an inconsistent background (e.g., a can of paint in a bedroom).

While our visual environment is rich and complex, it also has regular and redundant spatial structure that reduces some of this complexity. In addition to object co-occurrence, the local layout of objects can also guide attention. The local layout constrains the probability of finding an object at a certain location relative to a reference object. For example, when searching for a keyhole, observers tend to first inspect the areas below any doorknobs. Such regularities are commonly referred to as spatial dependency between objects. Spatial dependency between objects in a scene arises because: (1) some objects fulfill a function together that requires a specific spatial arrangement, for instance, a computer mouse is likely located to the right of a keyboard; (2) some objects are physically supported by other objects, such as a computer monitor on a desk; and (3) real-world scenes have syntactic structure; for example, the sky usually appears in the upper half of an image, and pedestrians often appear in the middle of an image on a sidewalk or crosswalk.

**FIGURE 5:**
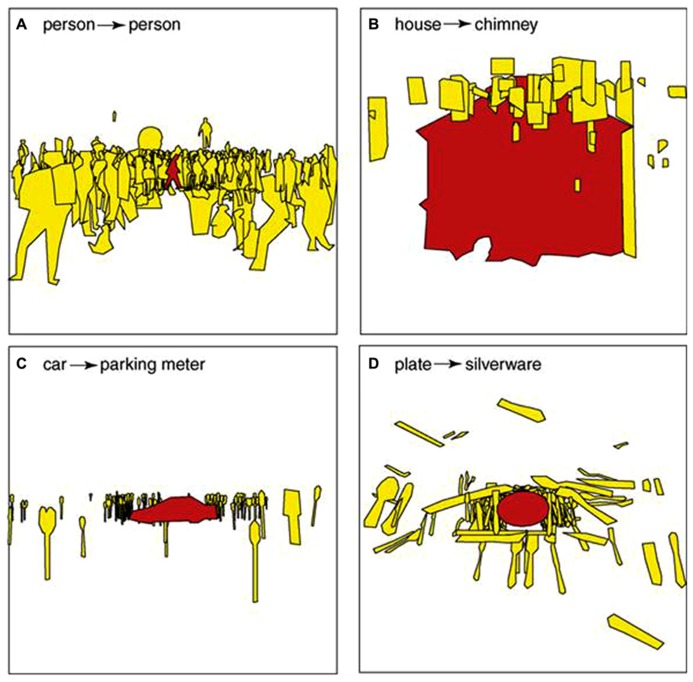
**Illustration of spatial dependency among objects.** Without being directly fixated, the identities and locations of target objects (shown in yellow) are conditional to the recognition of a reference object (shown in red). The reference objects are **(A)** a person, **(B)** a house, **(C)** a car and **(D)** a plate. The inferred objects are **(A)** other people, **(B)** a chimney, **(C)** parking meters and **(D)** a silverware. Figure reproduced from Oliva and Torralba (2007).

Oliva and Torralba (2007) reviewed the effect of scene context extracted from spatial dependencies among objects. When an object was recognized in the scene, the identities and locations of other objects became highly constrained. **Figure 5** shows some examples of inferring objects based on a reference object. Before the reference object (red object) is recognized, other objects around it can only be inferred based on their visual features. For example, the yellow objects in **Figure 5C** could be any items with elongated shape. Once the reference object has been recognized as a car, the other objects are most likely to be identified as parking meters.

In their pioneering work, Chun and Jiang (1998) coined the term *contextual cueing* for a paradigm where synthetic stimuli composed of L’s and T’s in certain spatial configurations were repeated throughout the experiment. They demonstrated that search time to locate targets in repeated configurations were significantly shorter than in novel arrangements of elements (Chun and Jiang, 1998, 1999, 2003; Olson and Chun, 2002; Jiang and Wagner, 2004; Brady and Chun, 2007). Later Brockmole and Henderson (2006a,b) found a similar contextual cueing effect in real-world scenes. In real-world scenes, it was shown that the first saccade during a search process would reliably go toward the predictive location of the target embedded in a real-world scene even when the target was absent, e.g., toward the top of a house when the target was a chimney (Beutter et al., 2003; Hidalgo-Sotelo et al., 2005; Eckstein et al., 2006; Droll and Eckstein, 2008; Ehinger et al., 2009; Castelhano and Heaven, 2010). The implication of this line of research is that knowledge of spatial dependency among objects is acquired through experience and is a top-down mechanism affecting visual processes (Chun, 2000).

There is an ongoing debate on how knowledge of spatial dependency among objects and knowledge of scene gist is utilized. In particular, there is some controversy regarding whether scene gist and spatial dependency are separate sources of information or hierarchically organized. The possibility most supported in the literature is that retrieving scene gist leads to knowledge of spatial dependency of objects in the scene (see Tatler, 2009). However, recent studies (Kanan et al., 2009; Castelhano and Heaven, 2011) question this assumption by showing that search can be guided by learned spatial dependency of objects and object appearance even without consistent gist information. For example, Brockmole et al. (2006) showed that the association between a target and its local context can be learned and bias attention when the global context in unpredictable.

Desimone and Duncan’s (1995) Biased Competition Model proposes that attentional guidance and motor behavior in a scene emerges from competition among objects, moderated by both top-down and bottom-up processes. It is plausible to assume that associated objects can be processed together more easily than unrelated objects. Moores et al. (2003) found such top-down associative effects on the deployment of attention during visual search. They showed participants a display of four objects in which some objects were semantically related to each other (e.g., a motorbike and a motorbike helmet). The participants were asked to search for a target (e.g., a motorbike) before such a display was flashed briefly (presentation duration ranged from 47 to 97 ms). Their results showed that participants were able to recognize and recall more often those objects that were semantically related with the target than unrelated distractors. The authors speculated that the template of a target causes our visual system to activate templates of semantically related items. Belke et al. (2008), using a similar paradigm, also found that attention was preferentially attracted to those objects that were semantically related to targets and that perceptual load did not affect this bias.

The landmarks of the environment provide cues as to where attention should be deployed. Chun and Jiang (1998, 1999) demonstrated that participants could learn arbitrary configurations of targets and distractors that were repeated over epochs at a global level, that is, the arrangement of items or relative positions of targets and distractors. They also showed that such learning could take place at a local level, i.e., through the co-occurrence of novel objects in the display and even motion trajectories of items in the display. In fact, the literature in this domain supports the view that the local layout of objects within a global configuration plays a dominant role for selection and maybe sufficient to demonstrate many of the major properties of contextual cueing (Peterson and Kramer, 2001; Olson and Chun, 2002; Jiang and Wagner, 2004; Brady and Chun, 2007; but see Brooks et al., 2010). This is an intuitively credible claim because observers usually cannot perceive the bird’s eye view of a scene immediately and must either search through their visual environment serially or use associative knowledge of items in their immediate view.

Unlike scene gist or object-scene relations, which can be acquired almost instantly in early visual processing and require little attentional resources, we need to identify at least one object in order to perceive relative associations among objects. Therefore, co-occurrence and spatial dependency of objects are perceived later than either scene gist or object-scene relations. Even though using object-object relations for attentional guidance may require more time and cognitive resources than relying on scene gist or scene-object relations, it still seems to be a commonly used strategy that observers adapt during natural scene viewing. It is likely that scene gist and scene-object consistency may affect only the initial stage of viewing (Brockmole and Henderson, 2006a). Zoest et al. (2004) suggested that top-down influences require at least 100 ms after scene onset to affect saccadic guidance. In real-world situations, however, natural viewing behavior usually involves inspecting the same scene for several seconds. Once an object is recognized, humans can form the spatial structure of a scene from their memory to infer the likely location of other objects (see Hollingworth, 2012, for a review). Thus, using spatial dependency between different objects may be a more efficient way to continuously decrease uncertainty about uninspected scene objects and continuously update the current search strategy.

## CONCEPTUAL SEMANTIC ASSOCIATIONS BETWEEN OBJECTS IN THE SCENE

**FIGURE 6:**
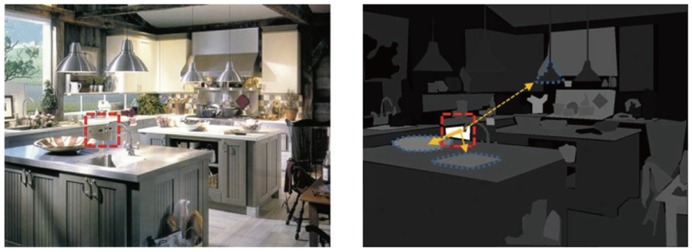
**An illustration of a semantic saliency map in an inspection task, as proposed by Hwang et al. (2011).** Left: the original scene. Right: The semantic saliency map based on the currently fixated object labeled “dishwasher.” The luminance of each object indicates how semantically similar it is to the dishwasher, as quantified by the corresponding LSA cosine value. The weight of the arrows indicates the likelihood of subsequent gaze transitions based on semantic guidance. The LSA cosine values for “bowl,” “sink,” and “hanging lamp” are 0.47, 0.39, and 0.14, respectively. Note that the values only indicate the relatively tendency for the subsequent gaze transition. They are not the probabilities.

As discussed above, many studies on the effect of semantic information on visual attention were based mainly on a single object-scene relation, which can be considered to be a simplistic approach. During natural scene inspection, semantic information may be continuously impacting observers’ viewing strategy, integrated with either low-level stimulus features or task goals. Therefore, any conclusions from studies using a single object-scene relation (either semantic or syntactic) might underestimate the use of semantic information in attentional guidance. Hwang et al. (2011) investigated how conceptual semantic similarity among scene objects influences attention and eye movements in real-world scenes. They asked observers either to view a natural scene and memorize its content or to search for a pre-specified target in a scene. In their experiments, each scene was selected from Label Me, an object annotated image data base (Russell et al., 2008) in which scene images were manually segmented into annotated objects by volunteers. They applied Latent Semantic Analysis (referred to as LSA; Landauer and Dumais, 1997) to measure semantic similarity between objects. Since annotated objects in Label Me have descriptive text labels, their semantic similarity can be estimated by computing the vector representations of object labels in a semantic space. Semantic similarity is then calculated as the cosine of the vector angle between object pairs, with larger values indicating greater similarity. Hwang et al. (2011) used this method to generate a semantic saliency map for each scene based on the semantic similarity of objects to the currently fixated object in an inspection task or the search target in a search task (see **Figure 6**).

Hwang et al. (2011) found that, during scene inspection, observers tended to shift their gaze toward those objects that were semantically similar to the previously fixated one. **Figure 6** illustrates this tendency: when the currently fixated object was a dishwasher, the next fixation was more likely to land on a bowl than on a sink, because the LSA cosine value was greater for the labels “dishwasher” and “bowl” than for the labels “dishwasher” and “sink.” Surprisingly, the use of semantic relevance between objects to guide visual attention, which was referred to as “semantic guidance,” still existed for transitions with long saccades of amplitudes exceeding 10° of visual angle. The authors showed that while the visual similarity between objects (semantically similar objects may share similar visual features) and their spatial proximity (semantically similar objects tend to be located close to each other) did contribute to the observed semantic guidance, the effect of semantic guidance did not disappear when both factors were ruled out. This finding implies that the role of peripheral vision in scene viewing is not limited to perceiving gist. Peripheral vision may also help in object recognition. This interpretation is supported by the results obtained by Kotowicz et al. (2010), who found that in a simple conjunction search task, the target was recognized before it was fixated upon. The function of the final saccade to the target was to simply increase the confidence of judgment. What is the function of semantic guidance? Observers may inspect semantically similar objects consecutively in order to quickly construct the concept for a given scene. For example, if the first few fixations are located on a pan, a stove, and a microwave oven, an observer may quickly develop the concept of a kitchen scene and also infer the likely appearance and location of other objects which are often found in a kitchen. The tendency of using conceptual semantic information may be an attempt to decrease memory load by grouping semantically similar objects so that the content of a scene can be encoded efficiently.

While performing a visual search task, participants in the Hwang et al. (2011) study tended to fixate on objects that were semantically similar to the verbally specified search target. This bias became more pronounced over the course of the search. It is possible that observers exploit semantic information in a scene for efficient search performance, leading to increased semantic similarity between fixated objects and the target as search progresses (Hwang et al., 2011). This finding is corroborated by Moores et al. (2003) and Belke et al. (2008), who found that, attention was attracted to an object which was semantically similar to the verbally specified target.

Interestingly, explicit assessment of semantic similarity between objects requires prior knowledge of their meanings. Consequently, attentional deployment would have to wait for the process of object recognition to completed, which seems to be an inefficient strategy of visual exploration. Thus, it is possible that the semantic guidance observed by Hwang et al. (2011) was facilitated by the use of observers’ knowledge about scene gist that they obtained in early visual processing. That is, instead of considering the semantic relation between the currently fixated object and the objects located in the extrafoveal visual field, observers can use their knowledge about the scene type to decide where to look next. For example, if observers were aware that the image was a kitchen, they may only attend to the regions nearby the counter or sink, where most of the – semantically related – kitchenware is likely located. This strategy could be executed by using the scene gist perceived during the initial glance without assessing semantic associations between objects (Oliva and Torralba, 2001, 2006). In addition to scene gist, observers could also obtain contextual information by exploiting the spatial dependency among objects and use it to predict the most likely location of a semantically related object or the search target (Oliva and Torralba, 2007). For example, a fork may be expected to be next to a spoon on top of a table. Therefore, if there was frequent gaze shifting from a table to a chair in a natural viewing task, it is possible that the visual system used the semantic similarity as a cue to make this decision, leading to the observation of semantic guidance. On the other hand, knowing the meaning of the “chair” object may not be necessary for making this transition. It is possible that when the table was fixated, the identities of other objects near the table were highly constrained. That is, the presence of the table limits the probabilities of other objects to appear next to it; they are very likely to be chairs or other furniture that is typically located near a table. Consequently, the decision of fixating on the chair was not necessarily made as a result of identifying it beforehand. The visual system may instead have chosen to fixate on a location where the possible occurrence of objects was highly constrained and thus contained the least uncertainty. Fixating on a predictive location may help recognize the object faster than fixating on a location with greater uncertainty. Other studies found a similar strategy in visual search tasks in which visual attention can be reliably directed to the predictive location of the target embedded in real world scenes even when the target was absent, e.g., toward the top of a house when the target was a chimney (Hidalgo-Sotelo et al., 2005; Eckstein et al., 2006; Droll and Eckstein, 2008; Ehinger et al., 2009; Castelhano and Heaven, 2010). Altogether, both scene gist and the spatial dependency among scene objects could contribute to the observed effect of semantic guidance without the need to identify extrafoveal scene objects. Further studies are needed to clarify whether the semantic guidance effect is due to the actual evaluation of semantic relevance between objects.

## CONCLUSIONS

It is well known that semantic information in natural scenes can influence attentional guidance. Research in this field has addressed a variety of different aspects of information retrieved or even inferred from the scene. These aspects are often generalized as a single concept such as semantic or contextual information from the scene. The current review attempted to disentangle the major semantic factors that guide visual attention. In summary, semantic information can be contributed from scene gist, scene-object relations, spatial associations between objects, or the semantic similarity between objects. Though most of these factors have been extensively investigated in the context of attention deployment, the issues listed below are still not well understood:

### WHAT IS THE ROLE OF ATTENTION DURING THE INITIAL LEVEL OF SCENE PERCEPTION?

It is clear that attention is necessary when semantic information involves recognizing the meaning of an object. However, many prior studies have shown that some semantic information such as scene gist could be perceived within less than 100 ms, which is too fast for focal attention and saccadic planning. Therefore, instant pop-out scene perception may be achieved without attention. In contrast, other studies found that scene perception was impaired when attention was fully engaged to an unrelated, concurrent task. This suggests that perception of natural scenes indeed requires visual attention. Whether attention can be exempted at any level of natural scene awareness, or if it is even essential for coarse-level scene perception is still an ongoing debate.

### WHAT IS THE ROLE OF OBJECT RECOGNITION WHEN PERCEIVING SCENE GIST AND OTHER SEMANTIC INFORMATION?

As discussed in Section “Contextual Information – The Gist of a Scene,” previous studies have found that humans can perceive scene gist without recognizing any individual object. Nevertheless, this does not necessarily imply that object recognition has no impact on the perception of gist. To determine the category of a scene, recognizing a representative object may be more useful than evaluating some global properties of a scene or its spatial layout. For example, recognizing a bed in a scene is more informative than evaluating the coarse spatial layout of the visual information for inferring a bedroom scene. Moreover, object recognition is needed for accessing the scene-object, object–object, and conceptual semantic relations. That is, observers need to recognize the currently attended object to infer the locations and identities of other objects in the scene. However, it is currently unknown whether the object that is going to be fixated next is recognized even before a saccade is initiated. Although some studies found that observers used semantic similarity as a cue to guide their attention (Moores et al., 2003; Belke et al., 2008; Hwang et al., 2011), this does not imply that the identities of other objects have been confirmed before they were fixated. It is possible that the meaning of objects cannot be confirmed before they are fixated, but the likelihood of their occurrence in a given location can be inferred based on the information retrieved from the currently fixated object. This strategy may induce conceptual semantic guidance without knowing the identity or meaning of any objects before attending to them. Whether observers adopt this strategy instead of evaluating the meaning of objects located in the periphery needs to be further investigated.

### HOW DOES THE USE OF SEMANTIC INFORMATION CHANGE OVER TIME WHEN VIEWING A NATURAL SCENE, AND HOW DOES IT INTERACT WITH THE TASK GOAL?

Previous literature has demonstrated that we perceive scene gist faster than other semantic information or the identity of objects. In spite of this, the time course of perceiving different pieces of semantic information does not have to align with the order in which these types first affect attention deployment. In other words, perceiving scene gist first does not necessarily indicate that attention is influenced by scene gist earlier than by other semantic information. Since scene gist only provides some coarse information about a scene such as its category, it is likely that the visual system may not deploy attention to other locations until the first object is recognized in order to avoid shifting gaze too early. In addition, the use of different aspects of semantic information must be sensitive to the goal of observers’ behavior. For a given scene, the use of different types of semantic information can be prioritized based on different task goals. How the task goal influences perception and use of semantic information is still not well understood.

Note that we are not claiming these different aspects are isolated independent processes. In fact, they may be tightly coupled. For example, the functions of scene gist and spatial dependency among objects not only help in understanding the content of a scene but also facilitate the process of object recognition. By accessing scene gist and spatial dependency among objects, people are able to infer the existence and location of other objects in a scene without directly fixating them. Therefore, when attention is guided to a new target, it may be difficult to determine whether this decision is made because the target has been recognized, or because the visual system was trying to decrease target uncertainty by using its knowledge about scene gist or spatial associations among objects.

The current state of attention models often considers each property extracted from a scene – such as faces, texts, or object luminance – as an isolated factor that can attract attention by itself. This approach seems incomplete, as it does not take into account the semantic associations between objects, arguably the main factor allowing human search performance in real-world scenes to still surpass modern computer vision approaches. Perhaps the relation between scene perception and different aspects of semantics can be described by the famous Gestalt notion: the whole is greater than the sum of its parts. Each component in a scene may contribute a different piece of semantic information, and these pieces can be treated as different variables that affect attention deployment. Nevertheless, the relations between these pieces may convey different types of semantic information (e.g., semantic consistency) which can be regarded as the interactions between different variables. In order to understand the whole perception of a scene and improve the search algorithms in current computer vision systems, knowing each part of its semantics and their associations is indispensable.

## Conflict of Interest Statement

The authors declare that the research was conducted in the absence of any commercial or financial relationships that could be construed as a potential conflict of interest.
